# Microfluidics for *in vitro* fertilization: from science to clinical validation

**DOI:** 10.1093/humupd/dmaf028

**Published:** 2025-12-05

**Authors:** Virginia Pensabene, Federica Agate, Andreia Santos Miranda, Helen Mary Picton

**Affiliations:** School of Electronic and Electrical Engineering, University of Leeds, Leeds, UK; Dipartimento Di Biologia E Biotecnologie “Lazzaro Spallanzani”, Universita’ di Pavia, Università di Pavia, Pavia, Italy; Leeds Institute of Cardiovascular and Metabolic Medicine, University of Leeds, Leeds, UK; Reproduction and Early Development Research Group, Discovery and Translational Science Department, Leeds Institute of Cardiovascular and Metabolic Medicine, School of Medicine, University of Leeds, Leeds, UK

**Keywords:** microfluidics, ART, IVF, engineering innovation, regulation, clinical validation

## Abstract

**BACKGROUND:**

This narrative review analyses the development of microfluidic technologies specifically applied to the IVF treatment, and their translation into clinical solution.

**OBJECTIVE AND RATIONALE:**

Starting with an analysis of the latest scientific publications, the patent scenario and the current clinical trials were analysed aiming to identify the most developed applications, the challenges, and barriers for regulatory approval and clinical validation in different countries.

**SEARCH METHODS:**

Searches were completed in English, by using a combination of these keywords (exceptions are included in the text in the different sections): Microfluidic, IVF, Assisted, clinical, fertility, human fertility, women fertility, reproduction, pregnancy, Assisted Reproductive Technology. These were used for previously published reviews and scientific journal papers using PubMed (National Center for Biotechnology Information at the U.S. National Library of Medicine), and Google Scholar, limited to the last decade (2013–2025); for completed or ongoing clinical trials using Clinicaltrials.gov; for existing patents and intellectual properties commercialization using lens.org, and crosschecked on espacenet.com from 2000 to 2025.

**OUTCOMES:**

It is approximately 20 years since the design of the first microfluidic systems for IVF. In the last 5 years, there have been over 130 publications proposing new microfluidic solutions, with pre-clinical validation data in animal models and humans. Our analysis highlighted three main areas of development that are discussed in terms of trends and advancements in oocyte and sperm processing and handling; proposed solutions to support *in vitro* embryo development; and microfluidic-based approaches and techniques for cryopreservation and female fertility preservation. In the last 20 years, progression of the microfluidic technology and improvement of manufacturing processes have led to an exponential rise of patents (1405) where microfluidics is applied to different steps of the assisted conception cycle. However, of these innovative techniques, only a limited number have progressed to clinical validation (19 trials commenced since 2009) and these have focused primarily on microfluidic sperm sorting and selection with multiple trials investigating its effectiveness in enhancing sperm quality and fertilization rates, and microfluidic embryo culture systems, where additional research is still needed to establish benefits over traditional culture environments. The key barriers to adoption include the need for long-term clinical outcome data, standardization of results across various patient populations, and regulatory challenges. We summarize the pathways needed to ensure compliance with quality standards and regulations in different countries. This analysis evaluates the different clinical trial requirements and challenges for participant recruitment, as well as study design complexity, and the definition of achievable endpoints and establishment of appropriate control groups or comparators.

**WIDER IMPLICATIONS:**

Finally, this review highlights complementary technologies recently combined with microfluidics (e.g. automatic and artificial intelligence-powered imaging, *in situ* non-invasive metabolic sensing) which can guarantee a more precise and safe handling of biological samples, favour automation of sample processing (e.g. gametes), and provide new information and higher level of control of the laboratory techniques used by clinics to treat patients in the next 5–10 years.

**REGISTRATION NUMBER:**

N/A.

## Graphical Abstract

**Figure:**
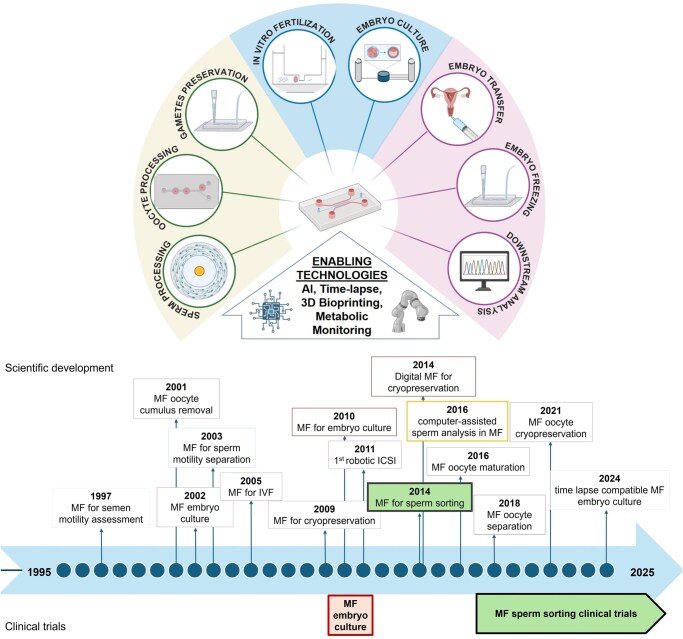
**Scientific development and adoption of microfluidic approaches in IVF since the early 2000s (MF, microfluidic; AI, artificial intelligence).** Schematic representation of the ART cycle, showing key applied microfluidic technologies, and enabling technologies, that could be soon integrated. The timeline at the bottom of the figure represents in gradual introduction of microfluidics in ART and in IVF. Created in BioRender. Pensabene, V. (2025) https://BioRender.com/4bh9ea5.

## Introduction

In the last 10 years, ART and IVF have been rapidly improving, driven by the exigency to offer larger opportunity of treatment to an increasing and diverse population and by the need to improve the clinical outcome of fertility treatments (Fang *et al.*, 2023; Graham *et al.*, 2023).

The ART treatment comprises multiple steps, each one being time consuming and meticulous, requiring high safety and efficiency. While improved scientific knowledge and clinical understanding of female and male physiology has perfected some of the steps involved, the success of many of these repetitive procedures is related to the individual skills of highly trained embryologists to ensure precise and timely handling of cells protects the cells from harm. The demands and use of advanced ARTs need to take into consideration the risk of human error as laboratory practitioners commonly work in a pressurized environment and there is considerable variability of practices and results between clinics (Lintsen *et al.*, 2010) and a general lack of standardization of protocols across clinics (Ma *et al.*, 2024). Automation, such as combination of robotics, microfluidics, and computing approaches, can be used to help reduce workload and costs and avoid risks (Angione *et al.*, 2015; Calhaz-Jorge *et al.*, 2020; Casciani *et al.*, 2021) while enhancing objectivity and outcomes in IVF labs.

The adoption of microfluidic technologies in ART has been proposed since the early 2000s, based on the many advantages that have been extensively proven in biomedical research and drug discovery (Figeys and Pinto, 2000; Sackmann *et al.*, 2014), such as the compact and drop-proof format of microfluidic devices, the reduced dimensions, with nl–µl volumes for fluids and cells, and the potential for high-throughput characteristics (Whitesides, 2006). In other applications, such as drug development, microfluidics is often combined with automated perfusion and robotic systems for the handling of liquids and cell constructs. These platforms are compatible with various imaging techniques and downstream omics analysis of sub-microlitre sample volumes. Microfluidic approaches also have the potential to ensure higher control, accuracy, and precision for monitoring complex processes when compared to standard manual processing or traditional dish-based culture protocols (Beebe *et al.*, 2002). Specifically in assisted conception, microfluidic technology may facilitate and improve several of the laboratory steps of the ART treatment (summarized in the graphical abstract), and thus to holds the potential to increase the overall success rates and to enable the development of more efficient and cost-effective patient treatments (Weng, 2019).

Here, we have reviewed the scientific development and application of microfluidic technologies to the field of ART over the 20 years since the first reports of microfluidic systems published in 2002 (Beebe *et al.*, 2002) and we discuss research progress and clinical uptake of microfluidics in ART as applied to sperm and oocyte processing, IVF, ICSI, embryo culture and embryo selection, cryopreservation, and female fertility preservation. Searches were completed in English, by using a combination of these keywords (exceptions are included in the text in the different sections): Microfluidic, IVF, Assisted, clinical, fertility, human fertility, women fertility, reproduction, pregnancy, Assisted Reproductive Technology. These were used for previously published reviews and scientific journal papers using PubMed (National Center for Biotechnology Information at the U.S. National Library of Medicine), and Google Scholar, limited to the last decade (2013–2025); for completed or ongoing clinical trials using Clinicaltrials.gov; for existing patents and intellectual properties commercialization using lens.org, and crosschecked on espacenet.com from 2000 to 2025. We have analysed the complexity of the translation pathways, starting from an analysis of the trend in intellectual property protection which represents the growth of innovative microfluidic concepts applied to ART. We then discussed the regulatory pathways and the challenges to meet numerous requirements and progress to clinical validation in different countries. Finally, we conducted a search on clinical trials and discussed the current and future combination of microfluidics with other technologies, such as artificial intelligence (AI)-powered imaging and *in situ* non-invasive metabolic sensing, that have the potential to further improve the success of ART treatments.

## Microfluidics applied to the ART cycle

### Sperm processing

Sperm sorting is an essential step in ART as the possibility to select good-quality sperm from a heterogeneous semen sample increases the fertilization, pregnancy, and live birth rates while reducing the risk of genetic abnormalities and unhealthy offspring (Zhang *et al.*, 2024). *In vivo*, sperm selection occurs naturally, with only highly motile and healthy sperm being capable of travel through the female reproductive tract and of reaching the oocyte in a timely manner. In clinics, good-quality sperm is selected based on multiple parameters that include motility, morphology, and DNA fragmentation. The commonly used methods of sperm processing (Henkel and Schill, 2003) include swim-up which is based on the evidence that sperm with chromatin maturity and enhanced morphology tend to swim against fluid flow, and density gradient centrifugation which exploits the higher density of motile and morphologically normal sperm cells compared to other cell types, dead cells, and cellular debris.

Numerous examples of microfluidic devices have been designed based on these principles (Aydin *et al.*, 2022) and extensively reviewed (Olatunji and More, 2022) (Table 1). Many of these systems aim to recreate the physiological and anatomical characteristics of the female reproductive tract (Ahmadkhani *et al.*, 2022) and demonstrated the ability to isolate a large number of high-quality sperm for fertilization with improved DNA integrity of up to 95% (Simchi *et al.*, 2021). Microfluidics has also provided new insights into the characteristic behaviours of animal (Nagata *et al.*, 2018) and human spermatozoa (Denissenko *et al.*, 2012; Ahmadkhani *et al.*, 2023).

**Table 1.** Microfluidic device for sperm processing.

Authors	Species	Microfluidic characteristics (manufacturing material; dimensions)	Objectives	Outcomes
Asghar *et al.* (2014)	Human	PMMA Channel width 1 mm; Chamber: Diameter 20 mm; Depth 3 mm;	− To investigate the motility of MF sorted sperm vs original sample	- Sperm motilities of ≥95% with MF vs initial 39.8%. - Sperm retrieval efficiency: saturated after 30 min 28.58%. - Maximum sperm retrieval efficiency with MF in 1:4 diluted sample: 52.68%. - DNA fragmentation (%): 1.1% with MF vs 31.2%± for unsorted semen.
Aydin *et al.* (2022)	Human	Fertile Chip	− to compare Fertile Chip vs swim-up technique	- Equivalent FR - IR: 50% with Fertile Chip vs 31% with SU. - PR: 62.5% with Fertile Chip vs 45.3% with SU; - CPR: 59.4% with Fertile Chip vs 35.9% with SU, - LB rates: 46.8% with Fertile Chip vs 25% with SU.
Ahmadkhani *et al.* (2023)	Human	PDMS; sinusoidal channels	− to investigate the synergic effect of rheotaxis and thigmotaxis on separated sperm quality	− Separated sperm exhibited 34.7% normal morphology, 100% motility, and 100% viability
Cho *et al.* (2003)	Human	PDMS; Channel: Width 100–500 µm Length 5000 µm Height 50 µm	−to demonstrate sperm isolation from samples with few motile sperm—no control	- 100% purity of motile sperm after sorting - Yields: 39–43% - improved sperm morphology
El-Sherry *et al.* (2023)	Human	PDMS, Single channel: Height 20 µm	− to evaluate the use of positive rheotaxis as a potential marker of male fertility and as a method for sperm motility analysis using CASA in MF channel	- positive rheotaxis % correlates with fertility - positive rheotaxis % decreases with sperm motility
Gonzalez-Castro and Carnevale (2019)	Stallion	FERTILE PLUS™ Sperm Sorting Chip	− to evaluate MF sorting to improve sperm quality and embryo development after ICSI (vs SLC or SU)	- Higher sperm motility, morphology, viability, membrane, and DNA integrity post-MF sorting vs SLC and SU. - MF sorting prior to ICSI did not affect cleavage and blastocyst rates.
Heydari *et al.* (2023)	Human	PDMS, Microchannels: Width 500–90 μm Height 100 μm	− to combines rheotaxis and boundary-following behaviour to separate sperm cells in terms of motility and swimming velocity	- 4 times increase in the volume of the processed sample compared with previous literature. - Isolated sperm are 100% motile. - Sorting based on swimming velocity. - Minimum injected flow rate for sperm reorientation: 1.5 μl/min.
Huang *et al.* (2014)	Human	PDMS coated with poly (ethylene glycol) methyl ether methacrylate (PEG-MA); Microchannel: Length 5000 µm; Width 400 µm; Height 70 µm.	− to enhance the human sperm motility sorting efficiency by laminar stream-based MF (no control)	Sperm viability: from 76.21% to 92.2% after sorting (assessed by flow cytometry).
Huang *et al.* (2017)	Human	PDMS coated with 1% bovine serum albumin, Microchannel: Length 5 mm; Width 0.3–0.6 mm Height not specified	− to enhance the human sperm motility sorting efficiency by laminar stream-based MF (no control)	Sperm viability: from 87.2% to 95.7% after sorting (assessed by flow cytometry).
Jeon *et al.* (2022)	Human	PDMS; First spiral channel (rectangular section): Width 800 μm Height 60 μm Second spiral channel (trapezoid section): Width 800 μm Height 80–120 μm	− to evaluate a multi-dimensional double spiral device with a check-valve-based recirculation method for sperm sample preparation	∼ 80% of sperm cell recovery; >99.95% removal of 10-μm beads representing leukocytes from low-viscosity semen samples, > 98% from high-viscosity semen samples; - ∼10 min for 50 ml of diluted semen sample; - continuous-flow separation; - sample volume processing: >1 ml of raw semen.
Kocur *et al.* (2023)	Human	ZyMot Multi (850 µl)	− to evaluate embryo euploidy rate utilizing a MF device for couples undergoing ICSI/PGT-A cycles (compared to DG)	- total SCF: 1.9% with MF, 26.2% in the raw specimen, 18.0% with DG; - dsDNA fragmentation: 0.3% with MF, 3.6% in the raw specimen, 3.1% with DG; - embryo euploidy rate: 42.9% in the MF cycles, 25.3% in DG cycles. - IR: 65.5% in the MF cycles, 6.7% in the DG cycles. - CPR: 64.6% with MF, 10.5% DG.
Kricka *et al.* (1997)	Human	Glass; Width: 100 μm Height: 40 μm Length: 10 mm	− to analyse sperm motility in a microchannel compared to conventional Makler chamber.	− Motility in the microchip correlated with forward progression of the sperm determined by the conventional Makler chamber method.
Lara-Cerrillo *et al.* (2024)	Human	ZyMōt™ ICSI	- to evaluate the impact of sperm DNA damage (SSB and DSB) on fertilization and embryonic euploidy after ICSI; - to study the impact of the MF device on fertilization and embryonic euploidy (DG centrifugation as control).	- FR: 70.84% using MF with normal SSB patients, 69.71% using MF with abnormal SSB patients, compared to 58.49% with DG. - Embryo euploidy rate: 60% using MF with normal DSB patients, 44.59% using MF with abnormal DSB patients, compared to 36.84% with DG. - number of euploid embryos increased using the MF with women >35 years old.
Li *et al.* (2018)	Human	Material not specified; 3 chambers: Radius>10 mm Channel: Heights and length>1 mm.	− to evaluate sperm selection performance of a MF combining chemotaxis and sperm progressive motility.	- sperm morphology: from 11.2% to 40.3% after selection, - DNA fragmentation: from 15.4% to 6.8% after selection.
Matsuura *et al.* (2013)	Porcine	Cyclic Olefin Polymer (COP), Dimensions not provided.	− to evaluate MF sperm sorting method for increasing sperm concentration in terms of time required for ICSI (compared to conventional micro-droplet method).	− MF reduces the average ICSI treatment time for poor-quality semen (265 ± 15 s vs 347 ± 19 s).
Nagata *et al.* (2018)	Bovine	PDMS; 3 communicating chambers (diameter 20–12–14 mm) Microchannels: Widths 0.5–0.6 mm Height 0.1 mm	− to evaluate MF for sperm sorting by rheotaxis through laminar flow	- MF sorted sperm has high DNA integrity and progressive motility. - sperm with transitional, sinuous patterns closely associated with successful fertilization outcomes and higher LB rates compared to rapid, linear progressive trajectory.
Nosrati *et al.* (2014)	Bull And Human	PDMS; 500 radial microchannels: Cross-section 100 μm×75 μm Lengths 6–7.5–9 mm	− to investigate the MF effect of time on selected sperm concentration, count, vitality, and DNA integrity.	- 1 ml of semen purified in under 20 min. - DFI 78% lower in the selected sperm compared to the raw semen sample; - 80% improvement of percent high DNA stainability (in 9 mm devices)
Panah *et al.* (2022)	Human	Material not specified; Microchannel: Width 40 µm; Height 50 µm.	- to evaluate MF efficacy for separation of motile/immotile sperm - assess viability after sorting	- motile sperm concentration: from 54.4 to 65.41 million/ml; - immotile sperm: from 30.66 to 21 million/ml; - live sperm viability: from 75.16% to 82.91% after sorting.
Phiphattanaphiphop *et al.* (2020)	Bull	PDMS; Width 200–500 μm Length 5 mm Depth 50 μm	− to optimize design specification for sperm separation channel	96% sperm sorting performance
Quinn *et al.* (2018)	Human	ZyMōt Multi (3 ml)	− to assess DNA damage after sorting morphologically normal sperm from a raw sample with MF (vs DG)	− DFI with MF 0%, compared to density-gradient centrifugation 6%, unprocessed sample 15%.
Quinn *et al.* (2022)	Human	ZyMōt ICSI Sperm Separation Device	to measure embryo quality after MF sperm processing for IVF with ICSI (vs DG)	No difference in high-quality cleavage stage embryo fraction, high-quality blastocyst fraction, clinical pregnancy, or ongoing pregnancy rates between groups.
Rahi *et al.* (2021)	Human	PDMS: 4-turns of the spiral microchannel: Width 70–150–200–300 μm	− to separate sperm cells from semen by inertial focusing combined with hydrodynamic filtration in multiple micro-slits (vs SU)	- sperm separation yield: 76% (with 88% purity in one-step); - 11.4% normal morphology percentage (4.8% unprocessed semen, 8.2% with SU method). - 4.6% DNA fragmentation (28.4% unsorted semen sample, 7.6% with SU;
Schuster *et al.* (2003)	Human	PDMS; Parallel channels: Width 100–300–500 µm Depth 50 µm	− to evaluate sperm motility separation by MF	- Sperm motility: 98 ± 0.4% from initial 44 ± 4.5%; - Morphology: 22 ± 3.3% from initial 10 ± 1.05%.
Seo *et al.* (2007)	Murine, Bull	PDMS; Multiple channels: Height 25 µm Lengths 23.46–8–5.5 mm Widths 205–100–80 µm	- to detect sperm DNA to enable sorting of X and Y chromosome-bearing sperm, - to separate sperm heads and tails for ICSI procedures	- motility rate 78.8% from initial 18.4%; - Average sorting rate (ea/min) 10.8
Simchi *et al.* (2021)	Human	PET Width 60 μm, depth 70 μm	− to facilitate sperm separation by mimicking epithelial characteristics in a MF (compared to SU and DG).	- max 98%%DFI in MF vs DG+SU outcome; - probability distribution function of B/A ≤ 1/3 for the MF compared to raw and DGC+SU samples - clinical usability (side-by-side): MF consistently selected sperm with <5% DNA fragmentation.
Suh *et al.* (2006)	Murine	PDMS; Width 500 μm, Depth 180 μm Length 2.25 cm	− to assess efficacy of MF for insemination, co-incubation, and fertilization vs conventional insemination in dish	- FR similar: 41% MF vs 42% control; - FR for denuded oocytes: 12% in MF vs 43% in control (10^6^ sperm/ml); 27% in MF vs 10% (8 × 10^4^ sperm/ml).
Wu *et al.* (2017)	Human	PDMS; Width 100 μm Height 100 μm Length 10 mm	− to evaluate a MF flowing upstream sperm sorter (control SU and DG)	–200 000 sperm/min -200 million cells/ml -90% of selected sperm are mobile; -∼20% of selected sperm has high motility (>120 μm/s). - 10% and 21% dead sperm for healthy and severe samples vs ∼50–60% with the SU or DG methods; - selection time: 10–15 min (2–4 h by SU; 1–2 h by DG)
Zaferani *et al.* (2018)	Bovine And Human	PDMS	− to demonstrate sperm separation by rheotaxis in MF	- MF enables to select minimum velocity threshold for isolated sperm. - normal progressive motility sperm (velocity: 48–93 μm/s for bovine, 51–82 μm/s for human samples) can be selected by adjusting the medium flow rate (0.6–1.8 ml/h).
Zaha *et al.* (2023)	Human	ZyMot-ICSI	− to evaluate FR, number, embryo quality, clinical pregnancy rate with MF vs traditional methods	- FR: no significant differences. - PR: 51% with MF vs 40% in control. - number of blastocysts: 2.66 with MF vs 1.63 in control.
Zeaei *et al.* (2023)	Human	PDMS; Network of boomerang-shaped microchannels: Width 200 µm Height 200 µm. Central channel: Height 200 µm.	− to evaluate MF for separation of highly motile sperm by inherent rheotaxis and boundary-following behaviour	- Sperm motility increased from 49% to 89% after separation; - sperm DNA fragmentation %: from 16% to ∼3% in selected cells

Studies including open microfluidics (e.g. microfabricated and/or communicating wells) and studies aimed to measure characteristics of the oocytes or understanding the effect of the microfluidic confinement on oocytes mechanical integrity were excluded from this list.

B/A, DNA halo width (B)/minor diameter of sperm head (A); CASA, computer-assisted sperm analysis; CPR, clinical pregnancy rates; CR, cumulus removal; DFI, DNA fragmentation index; DG, density gradient; DSB, double-strand breaks; dsDNA, double-stranded DNA; IR, implantation rate; LB, live birth; MF, microfluidic; PDMS, polydimethylsiloxane; FR, fertilization rate; PGT-A, pre-implantation genetic testing for aneuploidy; PR, pregnancy rate; SCF, sperm chromatin fragmentation; SLC, single-layer colloidal centrifugation; SSB, single-strand breaks; SU, swim-up.

The majority of the sperm microfluidic systems exploit the principle of:

1. Motility (Seo *et al.*, 2007; Huang *et al.*, 2014, 2017; Wu *et al.*, 2017) and rheotaxis (Zaferani *et al.*, 2018; Abdel-Ghani *et al.*, 2020; El-Sherry *et al.*, 2023; Zeaei *et al.*, 2023) when combined with computer-assisted sperm analysis. Achieving >90% efficiency with either human (Huang *et al.*, 2017; Wu *et al.*, 2017; Heydari *et al.*, 2023) or bull semen (Phiphattanaphiphop *et al.*, 2020), sperm microfluidic devices can generate controlled flow rates, where highly motile sperm swim and remain confined in microfabricated channels.
2. Chemotaxis (Gatica *et al.*, 2013; Li *et al.*, 2018; Yan *et al.*, 2021): flow and chemical spatial gradients can be precisely controlled in microfluidic devices to attract sperm responding to hormonal cues (e.g. progesterone) with a view to extraction of the highest quality fraction of each sample processed.
3. Thermotaxis (Bahat *et al.*, 2003, 2012; Boryshpolets *et al.*, 2015; Yan *et al.*, 2021): by recreating the natural behaviour of the sperm that is guided by the thermal gradient in the fallopian tube, microfluidic devices have been designed to select capacitated sperm.
4. Inertia focusing (Rahi *et al.*, 2021): microfluidic devices, mostly with single or double spiral shapes, show the ability to separate sperm from non-sperm cells in less than 10 min based on a multi-dimensional double spiral device with a check-valve-based recirculation method. This separation can be achieved without any sample pre-preparation or washing and is independent of the viscosity of the patient sample with∼ 80% of sperm cell recovery (Jeon *et al.*, 2022).
5. Custom made microfluidics devices have been also designed to exploit shear forces in a microfabricated channel for sperm separation and have confirmed lower DNA degradation with stallion and human semen (Panah *et al.*, 2022; Vigolo *et al.*, 2023).

Alternative platforms have also been developed to trap individual sperm cells and to select higher motility sperm using optical tweezers (Shao *et al.*, 2007). Paper-based assays or plastic-based devices for measuring sperm concentration have been developed into commercial solution to detect DNA sperm and for sperm count (Matsuura *et al.*, 2014, 2017; Nosrati *et al.*, 2016; Mirsanei *et al.*, 2022; Srinivas *et al.*, 2022) including SpermCheck Fertility Test, Babystart FertilCount Male Fertility test, FertilMARQ, FertilPlus, Qualis.

Recent studies of sperm samples with increased double-strand breaks (DSB) also provide evidence of improved outcomes using the microfluidic approach compared to conventional sperm processing methods, specifically with younger infertile patients (Lara-Cerrillo *et al.*, 2023). The use of a microfluidic device for sperm sorting, compared to density gradient centrifugation and swim-up, demonstrated significantly better clinical outcomes including: an increase of 28.3% in biochemical pregnancy rates; 35.6% in clinical pregnancy rates; and 35.3% in live births compared to the first ICSI cycle in couples where the male partner presented high levels of DSB in his sperm DNA (Lara-Cerrillo *et al.*, 2023). Furthermore, an 11.2% increase in fertilization rates has been demonstrated in patients with abnormal single-strand break (SSB) values accompanied by higher embryonic euploidy rates when a microfluidic approach was used, compared to couples whose sperm samples were processed with density gradient methods (Lara-Cerrillo *et al.*, 2024). Interestingly, the microfluidic approach has shown improved outcomes in cases of oligospermia, when, even with a lower sample concentration, a 37% fertilization rate was achieved (Suh *et al.*, 2006). Most of the microfluidic devices presented in the last 10 years, when compared to density gradient centrifugation methods for sperm, guarantee higher consistency, for higher blastocyst and birth rates in combination with ICSI (Kocur *et al.*, 2023; Zaha *et al.*, 2023). In contrast, a device-less microfluidic method that exploits Laplace and hydrostatic pressure laws for rheotactic sperm selection, significantly reduced the sample preparation for ICSI, avoiding washings, centrifugation, limiting the risks associated with handling, gamete mix-up and reactive oxygen species production (Bonduelle *et al.*, 1999). Given their potential for rapid isolation within 10–30 min (Nosrati *et al.*, 2014), microfluidic-based selection methods were deemed more efficient (Ma *et al.*, 2024; Zhang *et al.*, 2024) at producing healthy sperm and preserving DNA integrity (Matsuura *et al.*, 2013; Tsai *et al.*, 2016; Zaha *et al.*, 2023) by excluding the damage induced by centrifugation.

The rapid technological progression from the many animal studies to the application of microfluidic sperm processing to human clinical treatment sperm samples can be explained by considering two important aspects. The first is the deep interest and knowledge of engineers about the movement of spermatozoa over many centuries (Leeuwenhoek, 1679; Gadêlha *et al.*, 2020), which has facilitated accurate matching of the characteristics of the microfluidic environment (volumes, flow, density) to the known requirements for sperm motility. Secondly, pre-clinical and clinical testing have been facilitated by the higher access to—and availability of—human samples for research and by the higher number of cells per sample, as well as by the lower technical demands and training needs for the preparation and selection for clinical sperm use as compared to, for example, oocytes and embryos. The rapid uptake of microfluidic devices for sperm preparation has been enhanced by the development of the user-friendly, disposable, and single use systems that have been developed. Device size is limited, and is commonly comparable with histological slides so making their usage compatible with the most commonly used microscope in clinics. Thanks to the completely embedded volumes, microfluidic sperm devices also guarantee a more precise control of incubation conditions compared to gradient centrifugation methods and facilitate rapid processing that is compatible with the improved management of the whole ART protocol. Combined with the positive results obtained with animal studies (Gonzalez-Castro and Carnevale, 2019; Orsolini *et al.*, 2022; Vigolo *et al.*, 2023), this ease of use has favoured device progression to human trials and clinical studies and the translation into commercial products (Cho *et al.*, 2003; Schuster *et al.*, 2003; Nosrati *et al.*, 2014; Wu *et al.*, 2017; Nagata *et al.*, 2018; Samuel *et al.*, 2018; Mangum *et al.*, 2020; Godiwala *et al.*, 2022).

While the main goal of optimizing microfluidic sperm sorting is to provide a simple, fast, and efficient instrument for sperm sorting in IVF clinics, analytical approaches have also been developed as diagnostic tools for male infertility (Chen *et al.*, 2013a,b) or combined with other portable related technology (Kanakasabapathy *et al.*, 2017). The manufacturing and cost of these platforms, for example exploiting paper-based microfluidics, could favour their adoption in low-income settings (Martinez *et al.*, 2010; Yang *et al.*, 2017).

### Oocyte processing and fertilization

Accurate handling of oocytes is important in all assisted conception cycles and oocyte positioning is essential when preparing the oocyte for insemination by ICSI. These steps present challenges for the experienced practitioners, requiring specific training and set of skills to perform several manual steps in the shortest possible time.

During oocyte denudation following insemination by IVF, the expanded and mucified cumulus mass (Rienzi *et al.*, 2012), needs to be manually removed by repeated micro-pipetting during exposure to hyaluronidase enzyme, which introduces the risk of damaging the integrity and viability of the oocyte (Van de Velde *et al.*, 1997; Smith and Takayama, 2017). Microfluidic approaches have been proposed since 2001 as a vehicle for efficient cumulus mass removal by combining enzymatic exposure with induced flow through a narrow microfluidic channel to remove the cumulus cells (Zeringue *et al.*, 2001, 2005; Zeringue and Beebe, 2004) or by applying modulated acoustic radiation force into microfabricated wells to reduce the time taken for denudation (Mokhtare *et al.*, 2022). While the denudation efficiency of these microfluidic approaches is surprisingly high (98.6%, defined as percentage of completely denuded oocytes out of the total number of retrieved oocytes), and has proved to be efficacious with both IVF and ICSI, the resulting fertilization and blastocyst rates were shown to be equivalent to the manual denudation procedures (Weng *et al.*, 2018). Few studies were able to show improvement in terms of automation of the procedure, with shorter time, reduced handling steps and excluded detrimental effects on the integrity of the oocyte in comparison with equivalent manual procedures (Table 2).

**Table 2.** Microfluidic approaches for oocytes processing and fertilization, female fertility preservation, follicles, and oocytes culture.

Authors	Species	Microfluidic characteristics (manufacturing material; dimensions)	Objectives	Outcomes
**Oocytes processing and fertilization**				
Berenguel-Alonso *et al.* (2017)	Cow	COC	− to evaluate a MF device in COC for trapping, maturation and fertilization of bovine oocytes (dish as control).	- no significantly different oocyte maturation in MF vs dish; - sperm penetration %: 35.63% in MF vs 61.44% in dish; - Normal Fertilization (Male Pronucleus Formation): 78.8 % vs 82.9% in dish; - Abnormal Fertilization (Polyspermic Penetration): 21.9% vs 17%
Clark *et al.* (2005)	Pig	PDMS; Width: 1000 µm; Height 250 µm Length 38 mm	− to reduce the incidence of polyspermy after IVF by MF	- # Sperm/oocyte: 2.03 in MF vs 6.47 in controls; - Monospermic (% of penetrated): 55.42 in MF vs 22.33 in controls.
Iwasaki *et al.* (2018)	Cow	The microfluidic device has a 700 µm width, 1 mm height, and 10 mm long separation channel	− to separate high quality oocytes by density based separation in sucrose solution in MF vs conventional, morphological evaluation	− BR: 36% with MF vs 34% with conventional.
Zeringue *et al.* (2005)	Cow	PDMS; Restricted channel: Width 40 µm Height 400 µm	− to demonstrate cumulus removal by MF and embryo development (control traditional vortexing)	− CR increased development on Day 2 (∼20% to ∼356%) and BR at Day 8 (∼33% to ∼57%) when compared to vortex CR.
Weng *et al.* (2018)	Mouse	PDMS; Width 90–130 µm Height 275 µm	− to automate oocytes denudation and facilitate evaluation of oocyte quality and the injection of sperm	− no significant differences in FR or BR for IVF or ICSI when the oocytes are denuded on a chip or manually.
**Female fertility preservation, follicle, and oocyte culture and IVM**				
Aziz *et al.* (2017)	Human	MF in PDMS and PMMA; Gel for encapsulation: 0.2% sodium alginate Chamber: Diameter 6 mm Height 5 mm	− to evaluate the culture of a single human pre-antral follicles in MF vs dish	− Growth of ovarian follicles (hormonal trends and diameters increase) in MF not significantly different from stage matched controls.
Choi *et al.* (2014)	Mouse	PDMS; Core: 0.5% collagen; Shell: 2% oxidized or non-oxidized alginate; Channels: Height 80–200 µm Width 200–400 µm	− to use a MF to generate a microtissue in alginate and collagen for 3D culture of early secondary preantral follicles to fabricate the ovarian cortical and medullary tissues (vs 2D culture)	- preantral to antral stage development: 28% in 0.5%collagen/0.2% alginate vs 0% in oxidized alginate and in 2D; - ovulation: 100% with 0.5%collagen core+0.2% alginate shell in without LH and EGF treatment.
Del Valle *et al.* (2022)	Human	Polystyrene; Well diameter 5.5 mm Well height 1.2 mm Channel height 0.5 mm channel width 1 mm	− to evaluate dynamic MF to obtain functional mature oocytes from cryopreserved-thawed ovarian cortical tissue culture vs static culture	- follicular development in dynamic MF up to the secondary stage within 8 days with low efficiency. - 0.5 ml/min flow impacts viability of stromal cells in the ovarian cortical tissue.
Nagashima *et al.* (2018)	Cat and dog	Polymethyl methacrylate (PMMA) and double sided adhesive+1% alginate; Channels: Width 4 mm Height 254 μm Length 25 mm	− to use a dynamic MF for culture of follicles vs agarose gel culture system	- no significant differences in density of normal primary stage follicles (cat) or morphology (dogs) among treatment groups; - feline primordial follicles size: MF+2 µl/min flow 38.02 µm vs 40.42 µm in control; - canine follicles size: 28.84 µm in MF 10 µl/min; 32.18 in MF 2 µl/min flow vs 32.64 µm in control.
Thuwanut *et al.* (2022)	Mouse	PDMS; Microchamber (semicircular): Width 7 mm Height 1 mm	− to compare MF efficacies for ovarian tissue culture and *in vitro* follicle growth vs conventional dish.	- Primordial follicle (%): 54.7 in MF vs 55.1 in control; - Primary follicle (%): 28.9 in MF vs 26.8 in control; - Preantral follicle (%): 16.4 in MF vs 18.1 in control.
Oskouei *et al.* (2016)	Mouse	PDMS; Microchannel: Length 7 mm Width 140 μm Depth 200 μm Microchamber: Width 120 μm Length 120–900 μm	− to assess IVM in MF vs static culture;	- MII oocytes%: 83.04% in passive MF, 82/17% in active MF vs 55.35% in static system; - FR: 84% in passive MF, 88% in active MF vs 71.36% in static; - BR: 76.58% in passive MF; 84% in active MF vs 22.92% in static. - Glutathione levels in the oocytes: significantly higher in MF vs static.
Zargari *et al.* (2016)	Mouse	PDMS; Microchannel: Length 7 mm Width 140 mm Height 200 mm Square-shaped microchambers: Width 120 mm Length 120–360–600–900 mm	− to observe rapid loading, positioning, and maturation in MF (no control)	- efficient positioning; - spontaneous denudation; - evidences of maturation.
Xiao *et al.* (2017)	Mouse	3D printed resin; 0.5% alginate for follicles encapsulation	− to recreate in a MF platform the hormonal signalling and the endocrine loops between multiple organs for drug discovery and toxicology studies	- evidence of physiologically relevant hormonal patterns simulation; - evidences of individual ovarian follicle growth, maturation, ovulation, and granulosa cell luteinization

Studies including open microfluidics (e.g. microfabricated and/or communicating wells) and studies aimed to measure characteristics of the oocytes or understanding the effect of the microfluidic confinement on oocytes mechanical integrity were excluded from this list.

BR, birth rate; COC, cyclic olefin polymer; CPA, cryoprotectant agent; CR, cumulus removal; DFI, DNA fragmentation index; DG, density gradient; DSB, double-strand breaks; dsDNA, double-stranded DNA; EGF, epidermal growth factor; EWOD, electrowetting on a dielectric; GV, germinal vesicle; HR, hatching rate; IR, implantation rate; IVM, *in vitro* oocyte maturation; LH, luteinizing hormone; MII, metaphase II (MII) ovulation; MF, microfluidic; PDMS, polydimethylsiloxane; FR, fertilization rate; PGT-A, pre-implantation genetic testing for aneuploidy; PMMA, poly(methyl methacrylate); PR, pregnancy rate; SCF, sperm chromatin fragmentation.

As summarized in Tables 1, 2, and 3, most studies test the efficacy of new microfluidic devices using animal species, and as yet the technology has yet to be moved into validation with human oocytes (Zeringue and Beebe, 2004; Clark *et al.*, 2005; Weng *et al.*, 2018).

**Table 3.** Microfluidic devices for embryo culture and cryopreservation.

Authors	Species	Microfluidic characteristics (manufacturing material; dimensions)	Objectives	Outcomes
**Embryo culture**				
Alegretti *et al.* (2024)	Mouse, Cow, and Human	PDMS and Parylene; Chamber: Diameter 500 µm Channels: Height 30 µm Width 400 µm Pulsatile pump (0.1 Hz, 18 nl/min av. flow rate)	- to evaluate preimplantation embryo development in dynamic microfluidic culture compared to static culture in animal model. - complete a Phase I/Non-Inferiority Clinical Trial of Human Embryo Culture	- Murine: BR: significantly higher in MF vs in drops; birth rate and foetal development not statistically different. - Bovine: BR: 54% in MF vs 32% in drops; - Human: embryo quality: (≥8 cells/0% cellular fragmentation) 38 ± 0.4% in dynamic MF culture vs 27% static culture; -Day 3–5 blastocyst conversion rate: 77% with dynamic MF vs 44% in static culture.
Esteves *et al.* (2013)	Mouse	PDMS; 30 and 270 nl chambers	− to evaluate preimplantation development after MF culture	Group culture: - pre-implantation developmental rates up to 95% (4.5 days after fertilization), - birth rates: 30% comparable to conventional droplet culture. Single embryo culture: - birth rates: 29–33% in MF vs 20% in microdrop.
Heo *et al.* (2010)	Mouse	PDMS and Parylene; Funnel chamber: Diameter 100 µm; Channels: Length 1.5 mm Height 250 µm Width 1 mm Pulsatile pump (0.1 Hz, 18 nl/min av. flow rate)	− to evaluate preimplantation embryo development in dynamic MF compared to static culture microdrops	- Embryo development: HR: 71% dynamic MF, 23% static MF; 31% microdrops - Cell number: 109 dynamic MF, 60 static MF; 67 microdrops
Hickman *et al.* (2002)	Mouse	silicon-glass	− to evaluate preimplantation development in static or dynamic MF (0.1 and 0.5 µl/h) compared to static microdrops	- Development rate up to morulas: higher for static MF device vs microdrop; - BR: static MF and microdrops better than dynamic MF.
Kim *et al.* (2009)	Cow	PDMS+BSA coating; Width 160 µm Length 50 µm Height 200 µm	− to improve embryo development by recreating spatial restrictions in MF	− 8 cells cleavage rate: 56.77% in 160 µm channel, vs 23.97% in the straight channel
Mancini *et al.* (2021)	Mouse	PDMS Microchannel: Height 200 μm Width 250 μm Chamber: diameter 1250 μm	− to evaluate preimplantation development in static MF compared to static microdrops	- Day 5 BR: 90.48% in MF vs 86.40% in control; - HR: 32.18% in MF vs 46.61% in control; - Outgrowth rates: 18.2% vs 30.6% control; - Mean outgrowth size (μm): 144.5 μm in MF vs 142.1 μm control.
H. Y- Huang *et al.* (2015)	Mouse	Glass, ITO, Si3N4, Teflon, tape Microchannel: Height 260 μm Width 2 mm	− to compare the effect of dynamic culture in MF on preimplantation development and live birth rates after drop-let manipulation by EWOD in MF (control static MF culture)	- BR: 88.9% in dynamic vs 92.1% in static; - HR: 50 ± 9.8% in dynamic vs 21.2% in static; - LB rate: 50.6% from dynamic MF (no control).
**Cryopreservation**				
Gatimel *et al.* (2021)	Human	GAVI^®^ method (AV)	to assess inter-operator variability and clinical outcomes of semi-automated vitrification vs manual vitrification	Survival rate: 96% with manual vs 90% with semi-automated; Intact survival rate: 86% with manual vs 84% with semi-automated; Clinical PR: 27% with manual vs 22% with semi-automated. Inter-operator variability: no significant difference. Time: MV technique quicker than AV (minus 11 ± 9 min).
Guo *et al.* (2019)	Pig	PDMS; Length 1200 μm Width 1000 μm Height 150 μm	− to evaluate a MF to minimize Osmotic stress injuries with continuous CPA concentration change (control step-wise method)	- Oocytes survival rate: 95.3% with MF vs 79.4%in control; - Cleavage rate: 64.4% with MF vs 43.6% in control; - BR: 19.4% with MF vs 9.7% in control.
Lai *et al.* (2015)	Mouse and Cow	PDMS and glass; Exposure chamber/loading channel: Width 200 µm Height 150 µm	− to evaluate an automated MF device that minimize osmotic stress during CPA exchange for oocyte vitrification (vs manual protocol)	Murine model: - Max shrinkage rate: 7.51% volume/s with MF vs 4.13% with manual. - Cryosurvival rate: 100% for both MF and manual. - Blastomeres per blastocyst: 98 with MF vs 89 manual. - Bovine cytoplasmic lipid retention: 76.6% with MF vs 51.8% with manual;
Tirgar *et al.* (2021)	Mouse	PDMS; Exposure chamber diameter 2 mm	− to evaluate a MF to automate cryopreservation and minimize the osmotic shock to embryos (vs manual protocol).	- Blastocyst re-expansion rate: 96.07% with MF vs 97.4% in control; - HR: 38.8% in MF vs 44.4% in control.
Miao *et al.* (2022)	Mouse	PDMS; Square conical chamber: Height 2.5 mm Length 4–0.4 mm Width 1.2–0.3 mm	− to evaluate a MF to automate cryopreservation and minimize the osmotic shock to embryos (vs Cryotop method)	- Vitrification rate (GV and MII stage): 100% with MF vs ∼97% with Cryotop; - Survival Rate: GV stage: 97.4% with MF vs 94.4% in control, MII stage: 97.8% with MF vs 97.8% in control.
Pyne *et al.* (2014)	Mouse	Glass, ITO, Parylene C, Teflon, Cr, tape; Height 100 µm;	− to evaluate digital MF for manipulation and automated cryoprotectant concentration gradient generation (vs manual method and control not vitrified)	- Survival Rate: 77% with MF, 73% with manual, 100% in control. - Development Rate: 90% with MF, 91% with manual, 93% in control.

Studies including open microfluidics (e.g. microfabricated and/or communicating wells) and studies aimed to measure characteristics of the oocytes or understanding the effect of the microfluidic confinement on oocytes mechanical integrity were excluded from this list.

BSA, bovine serum albumin; COC, cyclic olefin polymer; CPA, cryoprotectant agent; EWOD, electrowetting on a dielectric; GV, germinal vesicle; HR, hatching rate; IR, implantation rate; ITO, indium tin oxide; LB, live birth; MF, microfluidic; MII, metaphase II (MII) ovulation; PDMS, polydimethylsiloxane; FR, fertilization rate; PR, pregnancy rate.

Translating and integrating the new microfluidic devices into the current clinical workflow has not yet proved possible, as to date most of the validation studies have been completed in animal models and the limited advantages recorded in pre-clinical studies also require the use of additional specialist equipment. Currently, there is still limited evidence of improvement of denudation efficiency or increased fertilization rates compared to standard procedures, both of which are needed to support the adoption of the new technology into clinical practice and to justify the higher costs and need for automated perfusion and/or additional equipment for implementation (Thapa and Heo, 2019). In initial evaluations of safety, microfluidic devices were used to measure the deformation and damage induced by shear stress generated in a microfluidic conduit (Luo *et al.*, 2015) and to measure the mechanical characteristics of oocytes using a combination of piezoelectric actuators and a robotic platform (Sakuma *et al.*, 2013; Nakahara *et al.*, 2015).

Microfluidic insemination of oocytes *in vitro* has been successfully demonstrated in murine models showing increased fertilization rates with lower semen concentrations in microchannels (8 × 10^4^–2 × 10^4^ sperm/ml) (Suh *et al.*, 2006), compared to standard of 1 × 10^6^ sperm/ml (Hogan *et al.*, 1994). As demonstrated in Table 1, microfluidic insemination has been successfully moved into validation with human oocytes and has now been adopted into clinics based on the improvement of efficiency for sperm processing. Interestingly, the microfluidic insemination approach mainly shows equivalent results to conventional methods when tested with murine and bovine animal models. These observations can be attributed to the biological characteristics of the samples analysed that lead to high blastocyst rates and overall fertility rates compared to humans due to factors such as, for example, faster embryo development rates and shorter gestation periods in rodent species. Improvements in outcomes become more quantifiable when validations are performed in humans, not only in terms of higher efficiency when processing samples, but also in terms of reduced sperm DNA fragmentation that are associated with higher fertilization rates in combination with ICSI, reduced embryo aneuploidy, and higher pregnancy rates.

The utilization of microfluidic systems could potentially reduce the complexity of the protocols used for inseminations by ICSI. This prospect holds promise even in cases of oligospermia. Thanks to laminar flow, in specialist microfluidic devices sperm can be predictably directed to each oocyte using a microchannel, whereas in traditional culture dishes sperm move rapidly and randomly in all directions (Suh *et al.*, 2006). Given the widespread use of ICSI in assisted conception clinics, the combination of ICSI with precise microfluidic push/pull procedures for the denudation of cumulus–oocyte complexes has been attempted, proving the feasibility of the concept and enabling improved visualization of the oocyte cytoplasm and oocyte orientation (Zeringue *et al.*, 2005; Lu *et al.*, 2011).

Advances in automation combined with reduction in the time taken for cell positioning and injection of oocytes during ICSI have been presented by McLennan *et al.* (2024). This research has shown that micro-ICSI led to a 56% improvement in the blastocyst rate for micro-ICSI injected porcine oocytes compared to conventional ICSI. However, while the microfluidic approach improved procedural efficiency and blastocyst rates, further optimization is needed to ensure a smoother workflow by improving alignment of injection height with sperm drops and minimizing loading/unloading time for oocytes. While this study includes a promising pilot with human oocytes, the approach needs more extensive validation and assessment of fertilization outcome, embryo quality, and live birth rates, to confirm clinical benefit.

While the evidence to date remains limited, there is clear promise of the use of microfluidic approach with oocytes. The advantages for this cell type being centred around higher precision and control combined with a reduction in the detrimental effects of the handling procedures on the mechanical integrity of the oocytes.

### Embryo culture and selection

The fundamental motivation to adopt the microfluidic technology for embryo culture and selection lies in its potential to go beyond the optimization of the chemical composition of the culture media (Swain and Smith, 2011) by introducing physical and fluidic stimuli that mimic *in vivo* requirements while facilitating the positioning and monitoring of individual embryos for non-invasive monitoring during development *in vitro*. Indeed, microfluidics allows practitioners to exert physical control over the embryos’ microenvironment, by confining the embryos within microcompartments of different shapes and sizes (Zeringue and Beebe, 2004; Heo *et al.*, 2010; Krisher and Wheeler, 2010; Esteves *et al.*, 2013) so more closely recreating the physiological and anatomical characteristics of the natural environment of the early embryo in the oviduct. Initial studies evidenced the negative effect of shear stresses and osmotic shifts on the embryo health (Heo *et al.*, 2007). However, progresses in device modelling and *in silico* simulation and new understanding of the mechanical integrity of embryos have allowed the design of new devices which exclude the detrimental effects of the shear stress generated during loading and retrieval on the development of embryos (Mancini *et al.*, 2021).

The substantial reduction of volume during microfluidic embryo culture and the confinement of one or more embryos into a sub-microliter volume of medium compared to conventional culture methods was initially questioned (Paria and Dey, 1990). However, several studies with animal and human embryos have confirmed the growth benefits of micro-volume culture through the accumulation and increased concentration of autocrine and paracrine factors in the media due to the close proximity of the embryos (Thouas *et al.*, 2003). Culture of single embryos or groups of embryos have been compared using number of different microfluidic devices (Table 3). Thanks to the possibility of precisely controlling the reciprocal position of the embryos within microfluidic chambers, it has proved possible to define the optimal ratio between number of embryos and culture volumes and thus to reach the optimal distance to facilitate paracrine signalling between embryos (Heo *et al.*, 2010; Esteves *et al.*, 2013). Concerns have however been raised about the impact of reduced culture volume on the risk of detrimental waste product build-up, oxygen deprivation, and nutrient depletion leading to lack of metabolic support for the embryos at the different stages of development when grown in the small volume of media that typify microfluidic culture (Heo *et al.*, 2010). These concerns have been addressed using innovative approaches with animal and human embryo models (Alegretti *et al.*, 2024).

Control of oxygen tension in microfluidic devices is possible (Rivera *et al.*, 2019), and can be accommodated both through the design of the device used and by the selection of the manufacturing material, which allow improved modelling of the typical physiological gas concentrations in the oviduct or uterus (as appropriate to embryo stage) needed to maintain the preferred 5% oxygen concentration used in embryo incubators in IVF clinics. Alternative approaches can also be used to set up the active perfusion of oxygenated media, or to modify the media composition with oxygen-producing or oxygen-scavenging chemicals (Palacio-Castañeda *et al.*, 2022). While the control of oxygen tension is accurate, the need for active perfusion systems, such as pumps, the increased complexity of the manufacturing process and the potential negative impact of waste products in the embryo secretome combined with potential toxicity of leachable compounds from the polymers and plastics used for device fabrication have limited the implementation of microfluidic embryo methods in human assisted conception (McLennan HJ *et al.*, 2024).

The implementation of dynamic culture conditions as a means to ‘refresh’ nutrients and eliminate toxic embryonic waste products such as ammonia has been proposed as a way to recreate the dynamic conditions of the oviduct *in vivo* and has been shown to have a positive influence on embryo development (Fauci and Dillon, 2006). As summarized in Table 3, dynamic culture have been explored using gravity/pressure driven media perfusion, by pulsatile, peristaltic, and passive pumping of media in the microcompartments (Hickman *et al.*, 2002; Walker and Beebe, 2002; Heo *et al.*, 2010; Esteves *et al.*, 2013), by mechanically tilting the whole microdevice (Kim *et al.*, 2009), and by displacement of the medium droplets using electrowetting on dielectric EWOD (Huang *et al.*, 2015). While continuous medium flow or medium refreshment affects embryo development and reduces viability and blastocyst rate (Hickman *et al.*, 2002; Kim *et al.*, 2009), punctual (Esteves *et al.*, 2013) or pulsatile medium delivery (Heo *et al.*, 2010) and periodic drop actuation (Huang *et al.*, 2015) have been shown to have a positive impact on embryo growth, implantation, and subsequent foetal development.

The validation and introduction of microfluidic approaches to embryo culture have been attempted since the beginning of this century, but their progression to clinical trials and clinical assessment remains limited. However, in the last 10 years, the impact of fabrication and manufacturing processes have been extensively analysed to better control gas exchange (Le Gac and Nordhoff, 2017) and to ensure chemical stability and safety of the plastics used (Palacio-Castañeda *et al.*, 2022). This scientific knowledge and technical advancement can now be applied to overcome the challenges for integrating the microfluidic culture into clinical protocols. In support of this idea, recent research has highlighted how some of the advantages of different microfluidic approaches could be exploited by reviewing and adjusting settings and protocols in the clinics (Swain and Smith, 2011), based on specific data and requirements available to implement these changes. For example, media composition, incubator dimensions and gas settings can now be adapted to meet the optimal requirements of humidity needed for a successful microfluidic embryo culture (Vajta *et al*., 2010). Similarly, the positive results obtained with active perfusion systems can be further explored and clinically validated by introducing new equipment such as pumps, controllers, and fluidic connections.

### Cryopreservation

Cryopreservation is routinely performed as part of assisted conception treatments (Wong *et al.*, 2014; Picton 2018) for different purposes that include:

- banking of sperm for patients undergoing infertility treatment and for sperm donors.
- banking and storage of embryos for planned frozen embryo transfers.
- banking of oocytes for patients at risk of ovarian hyperstimulation syndrome or cancer patients (Rodriguez-Wallberg *et al.*, 2020).
- for patients who wish to have elective oocyte freezing
- banking of sperm, oocytes, or embryos or gonadal tissues to safeguard the future fertility of oncofertility patients or patients at risk of fertility loss through chemotherapy treatments for non-cancer or genetic indications or as a result of trauma or surgery.
- preservation of healthy ovarian tissue for social reasons as source of oocytes for IVF attempts at later stage in life (Kasaven *et al.*, 2022).
- cryopreservation of isolated follicles for *in vitro* oocyte growth, maturation, and ovulation (Woodruff and Shea, 2007; Picton 2022) specifically important for patients with premature ovarian failure due to pelvic inflammatory diseases, ovarian endometriomas, polycystic ovary syndrome, or for cancer patients (Goswami and Conway, 2005; Salama and Woodruff, 2015).

Cryopreservation is carried out by either slow, equilibrium freezing or by ultra-rapid vitrification. While slow freezing requires precise, controlled freezing rates in the presence of relatively low concentrations of toxic cryoprotectant agents (CPAs) to prevent dehydration and intracellular ice formation (Mazur, 1970), vitrification is ice-free and requires the use of very high concentrations of glass-forming solutes and CPAs to avoid ice crystallization while the temperature is rapidly decreased. Propanediol and ethylene glycol along with sugars such as sucrose and trehalose are commonly used as CPAs to suppress ice nucleation, maintain the plasma membrane integrity and protein conformation in oocytes and embryos (Choi *et al.*, 2015; Huang *et al.*, 2015); glycerol is commonly used for sperm cryopreservation. While CPAs prevent ice-induced damage in the cells, these agents also introduce the risk of rapid osmotic pressure changes, and consequent swelling or collapsing of the cells. In contrast, microfluidic approaches to cryopreservation facilitate the dispense and mixing of liquid samples with nano (i.e. 10^−9^ l) to picometric (i.e. 10^−12^ l) precision (Squires and Quake, 2005) which can be used to establish a dynamic and precise perfusion environment unique to different cell types and sizes (Brody and Yager, 1997; Sackmann *et al.*, 2014). This is essential first to elucidate the osmotic and mass transport mechanisms of, for example, the embryonic membrane (Zhao *et al.*, 2017) but most importantly, it can also be used to precisely measure and control the loading of CPAs to reduce the volume changes which are critically important when preserving oocytes (Heo *et al.*, 2011).

A microfluidic cryopreservation approach was investigated by Lai *et al.* (2015) who exposed oocytes and zygotes to different CPA formulations and directly measured the response in terms of cell volume. The device was also used to study and measure the effect of each specific CPA included in the formulation. In Lai *et al.* (2015) and Tirgar *et al.* (2021) the volumetric changes of oocytes and zygotes were limited by generating either a temporal concentration gradient of CPAs in a microfluidic chamber and linearly increasing the CPAs concentration in a 0.377 mm^3^ volume chamber. Other groups have exposed embryos to different CPAs concentrations by moving them into different microdroplets (Pyne *et al.*, 2014), and by controlling the variation in CPA concentration in a square-conical-shaped microfluidic chamber with a capillary valve (Miao *et al.*, 2022).

The advantages of using a microfluidic device for human gamete and embryo cryopreservation have been previously reviewed and includes reduction of reagent consumption, decreased labour-intensity, ease of use, increased throughput, and improved survival rate after cancer treatment (Zhao *et al.*, 2017; Guo *et al.*, 2019), which become important with the increased demand for embryo cryopreservation in patients undergoing assisted conception (Bosch *et al.*, 2020). Recently, Gatimel *et al.* (2021) used a semi-automated vitrification method which combines a millifluidic pod with an automated injection/liquid handling system and showed no significant differences in outcomes as well as similar interoperability and time for complete vitrification compared to conventional vitrification methods.

The translation and clinical adoption of microfluidic cryopreservation methods requires further research to confirm efficacy and performance. In this future, this approach will be enhanced by an improved ability to leverage flexible manufacturing processes that incorporate high thermal conductive materials into microfluidic devices. This potential may be combined with novel, controllable, mineral ice nucleators (Daily *et al.*, 2023) as a means to improve the control of ice formation within microfluidic devices which will facilitate the automation of freezing processes in ART. Microfluidic cryopreservation may in the future provide a viable alternative to conventional freezing methods for the cryopreservation of different reproductive cell types with different freezing optima.

### Female fertility preservation, follicle, and oocyte culture

Existing approaches to female fertility preservation in girls and young women involve cryopreserving embryos, mature oocytes, or ovarian cortical tissue. In contrast, fertility preservation options in men include sperm freezing, while for prepubertal boys, the experimental technique of testicular tissue cryopreservation is advocated (Picton *et al.*, 2015). While autotransplantation of the preserved ovarian cortex is the proven way of restoring fertility in female oncofertility patients, this approach is not suitable for patients who were diagnosed with blood-borne cancers or who have a high risk reseeding their original disease by regrafting their ovarian tissue. These individuals must look to the development of a new research strategy for the complete *in vitro* growth (IVG) of follicles and subsequent cytoplasmic and nuclear maturation of the oocytes so derived from their banked ovarian tissue as the route to fulfil their baby wish. However, the IVG of follicles and oocytes is particularly challenging due to the difficulties of recreating the dynamic and complex endocrine, growth and nutritional environment *in vivo* needed to complete folliculogenesis and to produce healthy, mature, and fertile oocytes (Picton 2019; Gargus *et al.*, 2020; Picton 2022).

Microfluidics has been proposed as a means to address the challenges of long-term follicle culture that is needed to support the IVG and IVM of human oocytes. In support of this concept, early studies have demonstrated that biomimetic, microfluidic encapsulation of ovarian microtissue containing mouse preantral follicles for 3-dimensional culture is possible (He 2017) and that human and mouse preantral follicles can be cultured for up to 8–10 days in microfluidic chambers supported by calcium alginate beads or hydrogel shells (Choi *et al.*, 2014; Aziz *et al.*, 2017). This research is encouraging and showed equivalence in follicular growth and steroid production in the microfluidic chip-grown follicles compared to similar sized follicles grown using conventional IVG methods. In contrast, while dynamic culture under controlled shear stress in microfluidic conduits was not sufficient to show significant advantages over conventional methods for the culture of isolated follicles or follicles *in situ* within ovarian cortex in other animal models such as cats and dogs (Nagashima *et al.*, 2018) a more complex multiorgan, millifluidic system developed by Xiao *et al.* (2017), has facilitated the 28-day culture of mouse ovarian tissue explants by superimposing a specific hormonal stimulation pattern that supported murine follicle development to ovulatory stages *in vitro*.

A general concern of all microfluidic culture systems is its effects of fluidic and mechanical stress on cells or tissues. The impact of fluid dynamic stress on cryopreserved-thawed human cortical tissue has been explored (Del Valle *et al.*, 2022). Although dynamic culture enables follicular development within human cortex up to the secondary stage within an 8-day timeframe, the effectiveness of the culture device remains constrained. High flow rates (0.5 ml/min) cause elevated apoptosis levels in stromal cells of the ovarian cortical tissue suggesting that quasi-static conditions better support *in vitro* early follicular growth compared to dynamic perfusion. The importance of a precise design and flow control inside the microfluidic conduits have been confirmed using a mouse model with a pump-free microfluidic device for ovarian tissue culture and *in vitro* growth (Thuwanut *et al.*, 2022). In this case, the follicle development competency was maintained but still with reduced follicle morphology and comparable gene expression to static culture.

Other related research efforts have shown that the IVM of murine cumulus oocyte complexes over 24 h can be achieved using simple fluidic approaches for positioning and culture of groups of oocytes (Zargari *et al.*, 2016) and by implementing dynamic controlled perfusion (Oskouei *et al.*, 2016). These approaches demonstrated a spontaneous denudation of the cumulus complex due to the fluidic environment, without any enzyme treatment, and an increase in the number of MII oocytes that gave a higher rate of blastocyst formation post-fertilization. Furthermore, using microfluidic devices made of cyclic olefin copolymer (COC), a gas-impermeable material that supports the establishment of a hypoxic microfluidic environment, matured oocytes have exhibited reduced cumulus cell expansion but showed a higher percentage of fertilization (Berenguel-Alonso *et al.*, 2017).

Collectively, these early attempts at microfluidic IVG and IVM are encouraging, however, the complete *in vitro* production of human oocytes from primordial stages to maturity will require a far longer duration of culture combined with a far more sophisticated, multi-phase culture strategy than has been used to support murine IVG (Picton 2022). Further research is needed to determine in the early promise of microfluidic IVG in this advancing field translates into systems that support IVG oocyte production for therapeutic application.

## The process and challenges to progress from technological innovation to clinical validation

### The rise of intellectual property

In the context of ART, ‘speed to clinic’ typically refers to the amount of time it takes for a new product, in this case a microfluidic system, to progress from the initial conceptualization through the pre-clinical development, the clinical trials, and the regulatory approval to become available for use in a clinical setting.

**Figure 1.**
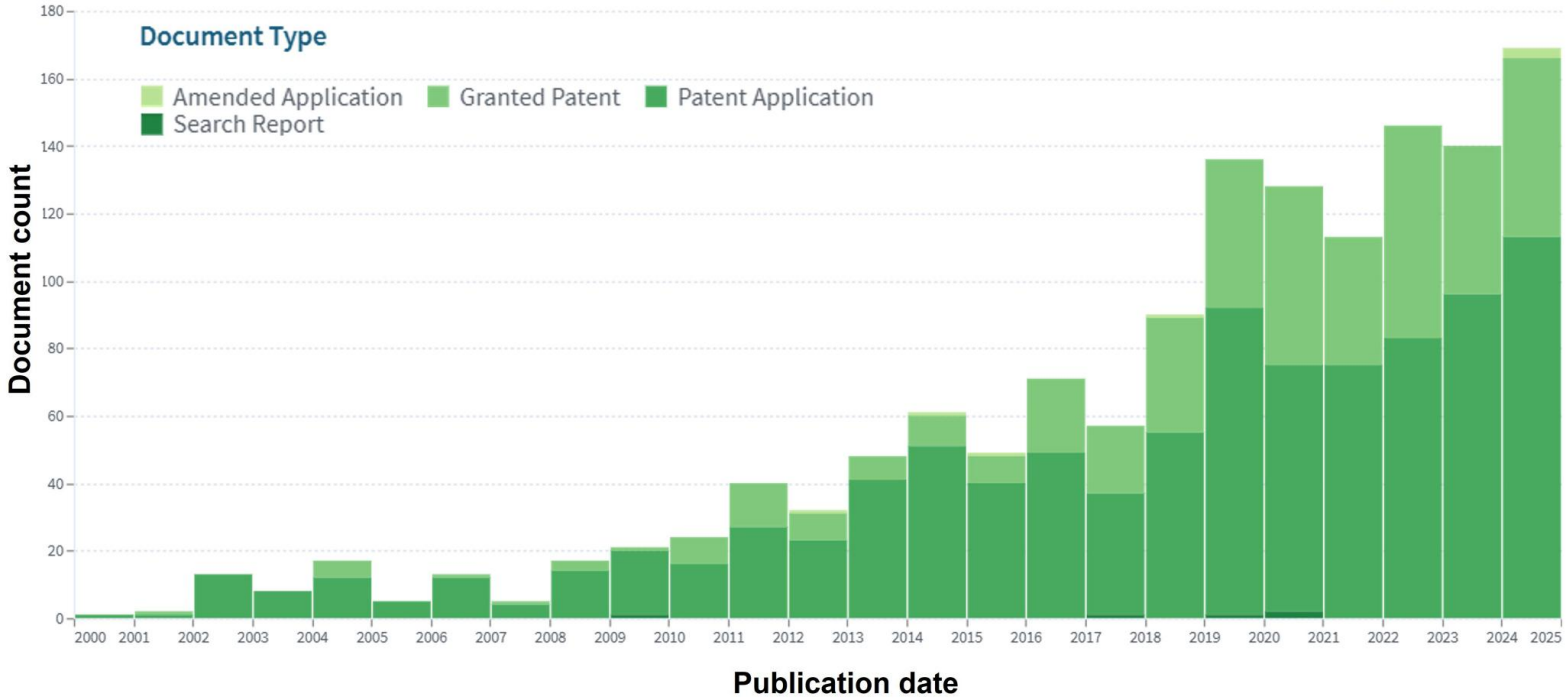
**The patent scenario.** Aggregated number of patents per year, grouped as Amended Applications, Granted Patents, Patent Applications, and Research Reports. Results of aggregated data collected from patent search on Lens.org on 15 January 2025, with keywords (IVF) AND (microfluidics) from 1 January 2000 to 1 January 2025.

Interestingly, since the early 2000s, innovative concepts based on microfluidic approaches have been gradually described by patents and growing over the years (Fig. 1).

**Figure 2.**
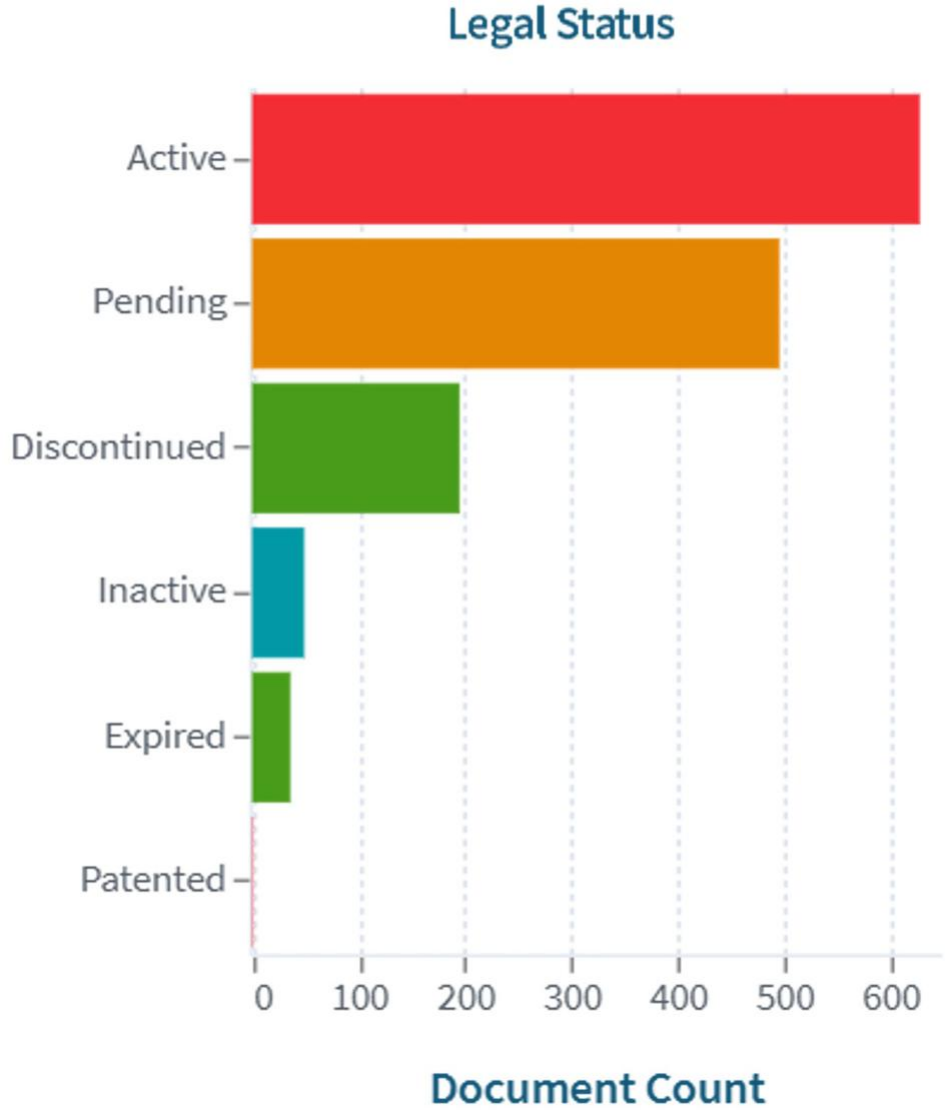
**Number of patents aggregated by legal status, either active, pending, discontinued, inactive, expired, or patented.** Results of aggregated data collected from patent search on Lens.org on 15 January 2025, with keywords (IVF) AND (microfluidics) from 1 January 2000 to 1 January 2025.

**Figure 3.**
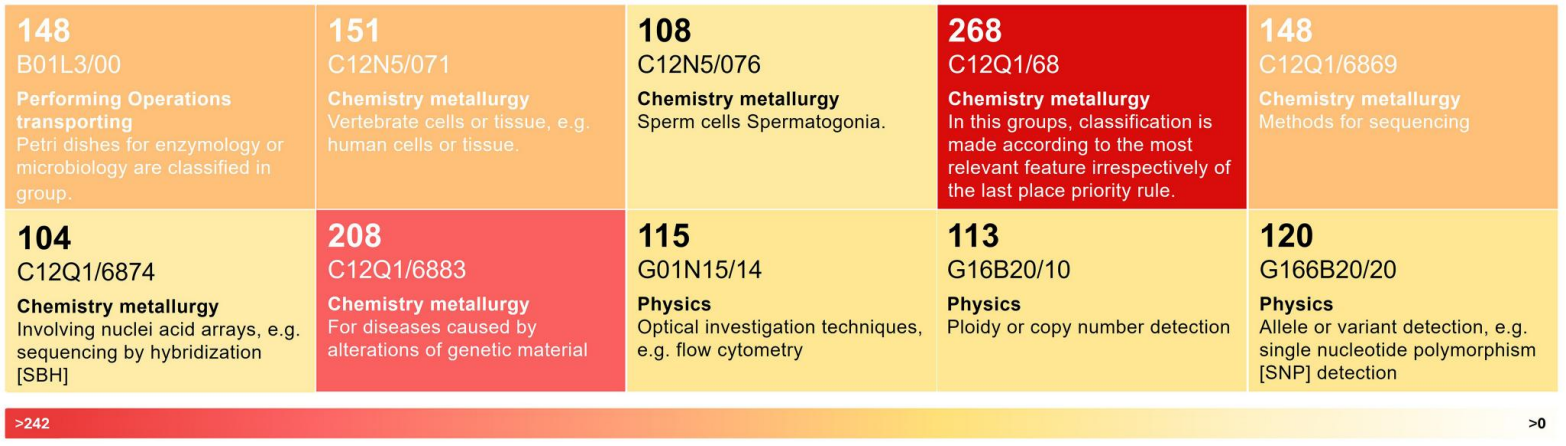
**Patents grouped by the most common Cooperative Patent Classification (CPC) code, indicating the category related to the technical subject matter.** Results of aggregated data collected from patent search on Lens.org on 15 January 2025, with keywords (IVF) AND (microfluidics) from 1 January 2000 to 1 January 2025.

**Figure 4.**
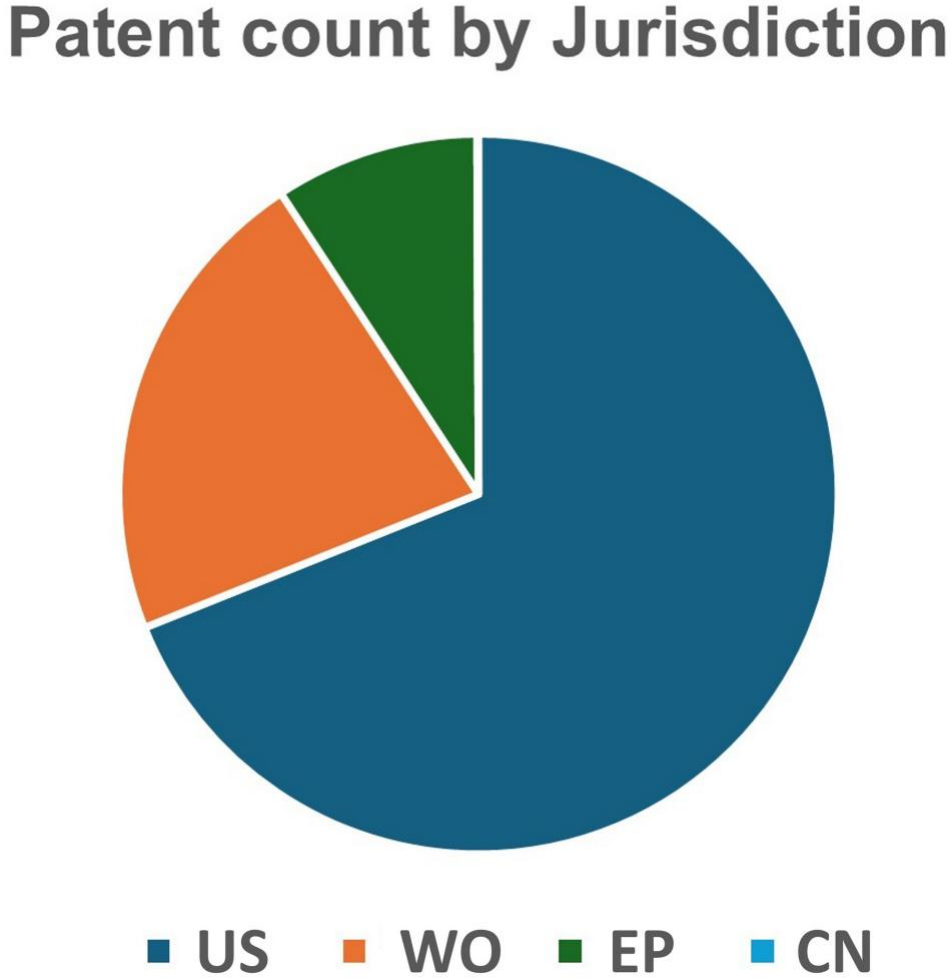
**Patent count for jurisdiction.** Pie chart aggregating patents by the most common jurisdiction where the patent offers protection: United States (US), Europe (EP, specifically the European Patent Office), and China (CN). Results of aggregated data collected from patent search on Lens.org on 15 January 2025, with keywords (IVF) AND (microfluidics) from 1 January 2000 to 1 January 2025.

Typically, the protection of the novel intellectual property precedes the publication in scientific journals and is essential for later commercial exploitation. From the analysis of the patent scenario, as summarized in Figs 1, 2, 3, and 4, we can derive several conclusions. The growing number of patents confirms the pace of innovation within the ART sector (Fig. 1), aligned with the availability of new and more accurate microfabrication processes and the increased knowledge in material properties. Migration from prototyping materials, such as polydimethylsiloxane (PDMS) or glass to thermoplastics like poly(methyl methacrylate) (PMMA) or COC demands redevelopment of surface treatments and bonding protocols due to differences in wettability, gas permeability, and mechanical modulus. Precision injection molding imposes stringent design-for-manufacture tolerances (e.g. global flatness ≤300 μm, ±5% feature control over 100 mm) and high upfront mold costs, leading to long lead times (3–5 years) and rigorous quality control (Cong and Zhang, 2022). Testing and validation present hurdles for thermoplastic microfluidics, that must undergo exhaustive extractables and leachables profiling (ISO 10993-18), cytotoxicity assays (ISO 10993-5/4), plus optical, thermal, and chemical stability testing under sterilization and accelerated aging. Manufacturing new and complex microfluidic designs, for example requiring multi-layer bonding or multiple materials, can exacerbate batch-to-batch variability, increase regulatory approval timelines, development costs, and ultimately time-to-market (Cong and Zhang, 2022).

This exponential growth is also supported by the cumulative interest of the clinical community, and the gradual availability of data on usage and performances of new prototypes. The analysis shows an inevitable arrest in the development of new technologies, demonstrated by a fraction of patents either discontinued or expired (Fig. 2). This fraction is however small compared to the overall number of patents that progressed and remained active (19.6%), and remained stable over time. This confirms a robust and active innovation environment, suggests a continuous and not decreasing interest in the clinical application. Considering the typical lifespan of patents in the main jurisdiction (USA), and the traditionally long average time necessary to obtain regulatory approvals in the ART field (see section below), the rate of patent discontinuation (with a total percentage of 13.6% on total patents in the 2020–2025 period) is relatively low and indicates that many technologies remain commercially relevant, reflecting sustained interest and investment in this area.

By aggregating patents with equal Cooperative Patent Classification (CPC) code (Fig. 3), the majority of the innovations are related to processes diagnostic methods based on measurement, testing, or detection of DNA (C12Q1/68 ‘family’), or focus on the manipulation and culture of cells, specifically human, sperm cells, or to their modification, encapsulation (C12N5). Additional patents follow in the ‘Chemical or physical apparatus for general laboratory use, particularly mixing or stirring apparatus’ classification (B01L30) or are related to bioinformatic methods or systems for processing genetic or protein-related data (G16B). Considering the multiple steps where microfluidics can be applied during assisted conception treatment cycles, sperm processing and analysis methods are the most crowded areas, together with the design of closed and confined culture of cells (i.e. embryos). The remaining areas, such as embryo transfer and cryopreservation, largely reflect the gaps in existing patents where new innovations could be developed with minimal risk of infringement.

In terms of competitive landscape, 69% of patents (including pending and granted, Fig. 4) are filed in the USA, 22% in the World Intellectual Property Organization (WO) and 9% in Europe. A limited number of Chinese patents is extracted by the analysis, which is due to the lack of translation of documents from the original language. The higher percentages in the USA are justified by the different approach to IP protection in North America, where multiple related inventions (i.e. multiple inventive concepts) can be included in one application. This picture also matched with the numerous scientific publications output of academic projects in American and European universities or summarizing clinical trials in these regions (as summarized in Tables 1, 2, 3, and 4). Investments in IP protection, maintenance, and expansion in these jurisdictions indicate active commercial interest and activity.

**Table 4.** Clinical study analysis.

Study title NCT number	Study status	Conditions	Interventions	Study type	Start date/completion date	Locations
Sperm selection using microfluidic technology (04997070)	Completed	Infertility, male	–	Observational	16 May 2022 to 1 March 2024	Belgium
Microfluidic chip vs density gradient (06005311)	Recruiting	Infertility	Device: microfluidic chip	Interventional	1 November 2024 to 30 November 2029	Hong Kong
Sperm selection by microfluidic separation (03085433)	Completed	Sperm DNA fragmentation	Device: microfluidic sperm sorting	Interventional	17 March 2017 to 30 April 2022	CA, USA
Embryo development in microfluidics system (01003548)	Unknown	Infertility	Device: smart embryo culture	Interventional	2009–2011	Brazil
Microfluidic chip vs density gradient (05978947)	Recruiting	Infertility	Device: Zymōt multi 850 µl	Interventional	1 July 2023 to 31 March 2025	Hong Kong
Microfluidics vs gradient centrifugation (04744025)	Unknown	Infertility, recurrent pregnancy loss	Device: microfluidic chamber	Interventional	13 January 2021 to 31 December 2022	USA
Effect of microfluidic sperm sorting (03960229)	Completed	Male infertility	Other: microfluidic sperm sorting chips	Interventional	23 May 2019 to 23 September 2019	Turkey
Microchip vs density gradient (04144244)	Unknown	Unexplained infertility	Other: microfluidic sperm sorting chips	Interventional	15 November 2019 to 15 July 2020	Turkey
Sperm separation efficiency (04061486)	Completed	Infertility, male	–	Observational	8 August 2019 to 9 January 2022	Spain
TESA vs microfluidics (05866484)	Completed	Male infertility, azoospermia	–	Observational	10 May 2023 to 31 May 2024	Canada
Sperm selection and embryo development (04962074)	Completed	Reduced sperm motility	Device: microfluidic sperm sorting chip	Interventional	5 January 2020 to 20 September 2020	Turkey
Microfluidic chip vs density gradient (06023472)	Recruiting	Infertility	Device: microfluidic chip	Interventional	1 November 2024 to 30 November 2028	Hong Kong
Sperm preparation for IUI (05693220)	Recruiting	Infertility, secondary infertility	Device: Zymot multi sperm separation	Interventional	5 February 2023 to 31 December 2025	San Francisco, USA
Live birth rate after Zymōt (06144268)	Recruiting	Infertility	Device: Zymōt multi (850 µl)	Interventional	10 January 2024 to December 2027	Belgium
Microfluidic sperm selection (03355937)	Unknown	Unexplained infertility	Procedure: sperm chip	Interventional	1 January 2018 to 31 December 2019	Turkey
*In vitro* human embryo culture (00985218)	Completed	Infertility	Device: smart system	Interventional	2009–2010	USA
Epic study: paternal age and sperm selection (06629766)	Not yet recruiting	IVF patients	Device: microfluidic sperm separation	Interventional	December 2024 to December 2026	USA
Characterization of sperm populations (05383599)	Recruiting	Male infertility	–	Observational	16 July 2022 to 1 August 2023	Belgium
Zymōt sperm selection and IUI (06384794)	Recruiting	Male infertility	Device: Zymōt sperm selection	Interventional	29 June 2023 to 31 December 2026	Spain

Clinical trials analysis completed in the US Clinical Trials registry (https://clinicaltrials.gov/, last search: 1 January 2025), using a combination of these keywords: Microfluidic, IVF, Assisted, clinical, fertility, human fertility, women fertility, reproduction, pregnancy, Assisted Reproductive Technology, filtered by ‘infertility’ as condition or disease, and ‘microfluidic’ other terms. Collateral information on these trials and the used systems was derived from published articles via a literature search.

### The complexity of the regulatory pathway

Clinical validation plays a crucial role in securing regulatory approval for medical devices intended for use in IVF clinics, with oversight from agencies such as the European Medicines Agency (EMA) and the Medicines and Healthcare products Regulatory Agency (MHRA) in the UK, the Food and Drug Administration (FDA) in the USA, and regulatory bodies across Asia, including China’s National Medical Products Administration (NMPA) and India’s Central Drugs Standard Control Organization (CDSCO). Despite regional differences in requirements, several common challenges complicate the approval process.

One of the most significant hurdles is the variation in clinical trial requirements across jurisdictions. In Europe, under the Medical Device Regulation (MDR), a Clinical Evaluation Report (CER) is typically required, often relying on real-world data or systematic literature reviews. This is valid particularly for Class IIa and IIb devices (medium or medium-to-high risk medical devices that are in contact with the body, respectively, for a short period of time). Examples of these are devices for sperm separation or embryo culture. However, high-risk devices (Class III) necessitate full-scale clinical trials.

The FDA’s approach differs according to the level of risks. For many devices, a 510(k) submission allows for approval based on predicate devices, eliminating the need for new clinical trials, whereas Class III devices undergoing the Premarket Approval (PMA) process must demonstrate safety and efficacy through extensive studies. In contrast, regulatory authorities in other countries (China or India) mandate local clinical trials for Class III devices, even if they have already secured FDA or CE approval. Japan’s Pharmaceuticals and Medical Devices Agency (PMDA) and South Korea’s Ministry of Food and Drug Safety (MFDS) may accept foreign clinical data, yet in some cases, local trials remain a prerequisite. As a result, manufacturers of new microfluidic devices are often compelled to conduct multiple, redundant clinical studies to satisfy various regulatory demands, leading to significant cost escalations and prolonged approval timelines (Table 5).

**Table 5.** Regulatory pathways.

Region	Regulatory authority	Key regulation	Approval process	Clinical data requirement	Average approval time (months)
USA	FDA	21 CFR 820 (QSR), 510(k), *De Novo*, PMA	510(k) if predicate exists; *De Novo* if novel; PMA for high-risk	Required for PMA; not always for 510(k)	9–24
EU (EEA)	Notified Bodies, Competent Authorities	MDR 2017/745	CE Mark via Notified Body	Required for Class III; literature-based for Class IIa	12–24
UK	MHRA	UK MDR 2002 (as amended)	UKCA Mark (MHRA+Approved Body)	Same as EU MDR	12–24
China	NMPA	Order 739 (Medical Device Regulations)	Class II: NMPA registration; Class III: Clinical trials+NMPA approval	Required for Class III; sometimes waived for Class II	18–36
Japan	PMDA	PMD Act	Class II: third-party review; Class III: PMDA approval	Accepted from foreign data but may need local validation	12–24
India	CDSCO	Medical Device Rules, 2017	Registration+Import License	Required for Class C; CE/FDA data may be accepted	6–12
South Korea	MFDS	Medical Device Act	K-GMP+MFDS approval	Accepted from CE/FDA, but local trials may be required	12–18
Singapore	HSA	Health Products Act	Expedited if CE/FDA approved	Usually waived for CE/FDA products	6–9

The regulatory pathway is summarized here in different countries, in terms of regulatory authorities (FDA—Food & Drug Administration, MHRA—Medicines & Healthcare products Regulatory Agency, NMPA—National Medical Products Administration, PMDA—Pharmaceuticals & Medical Devices Agency, CDSCO—Central Drugs Standard Control Organization, MFDS—Ministry of Food and Drug Safety, HAS—Health Sciences Authority) and key regulations for Class II and Class III devices. The corresponding approval process and the associated requirement to provide clinical data and comply with quality management standards affect the final overall approval time, here indicated in months.

Beyond trial requirements, patient recruitment poses a substantial challenge, particularly within the field of ART. The eligibility criteria for enrolling patients in fertility-related studies are stringent, often limiting participation based on age, fertility diagnosis, or prior treatment history. Moreover, ethical concerns surrounding embryo research necessitate extensive approvals from ethics committees, especially in regions where regulations are particularly conservative. In countries such as India and several Middle Eastern nations, additional restrictions are imposed on embryo-related studies, making trial design and execution even more complex. These barriers frequently lead to delays in study initiation and prolong overall research timelines.

Study design itself represents another layer of complexity. Regulatory agencies demand clinically meaningful endpoints, which, in the context of ART, may include improvements in sperm selection efficiency for ICSI, increased embryo viability rates, or higher pregnancy success rates. Some agencies, such as the FDA and NMPA, extend these requirements to include long-term neonatal health tracking post-birth to assess potential adverse effects associated with novel ART technologies. Defining appropriate endpoints that satisfy regulators while ensuring feasibility within the constraints of IVF research can again significantly extend study durations and increase costs.

Equally challenging is the need to establish an appropriate control group or comparator in all clinical trials. Regulators typically favour randomized controlled trials comparing the novel microfluidic device against a recognized gold standard. However, due to the novelty of many microfluidic technologies, an established benchmark may not exist, complicating study design and statistical analysis. Demonstrating superiority over traditional sperm sorting techniques or standard embryo culture methods, for example, is particularly difficult in cases where the incremental improvements provided by microfluidic devices are subtle yet clinically significant. Furthermore, achieving sufficient statistical power in ART-related clinical trials presents inherent difficulties (Wilkinson and Stocking, 2021). Given the relatively small patient pool undergoing assisted reproductive treatment at any given time, recruiting an adequate sample size for statistically significant results is challenging. Regulatory authorities often reject underpowered studies, requiring researchers to conduct additional trials, which further inflates study costs and extends approval timelines. This issue is exacerbated when attempting to stratify data across multiple patient subgroups, as required by some agencies.

Finally, data acceptance and harmonization across regulatory jurisdictions remain a persistent issue. While the EU MDR and UK MHRA may accept foreign clinical data in some instances, agencies such as China’s NMPA and India’s CDSCO require independent, locally conducted trials, irrespective of prior FDA or CE approvals. Even within the USA, the FDA may mandate supplementary validation studies tailored to the American patient population, increasing the complexity of global regulatory submissions. This lack of harmonization forces manufacturers to navigate a fragmented approval landscape, often leading to duplicated efforts and inefficiencies.

These challenges of securing regulatory approval thus explain the delays observed in the validation of microfluidic devices for embryo culture in comparison with sperm processing, due to complexity to comply with international regulatory requirements and limited availability of samples vs number of recruited patients in the first case.

### Completed and ongoing clinical trials

**Figure 5.**
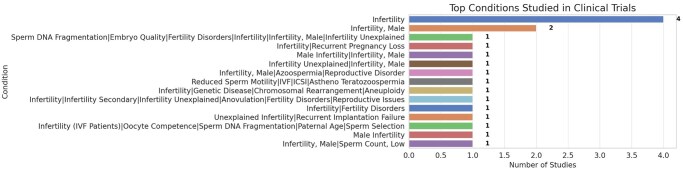
Top conditions studied in clinical trials: distribution based on conditions.

The scientific development, described in the literature and discussed above, has supported the progression of a limited number of microfluidic products into clinical validation and clinical use. By completing a systematic search for microfluidic systems in the US Clinical Trials registry we identified a total of 19 results (Fig. 5).

One of the most extensively studied applications, as discussed above, is microfluidic sperm sorting and selection, with multiple trials investigating its effectiveness as a tool to enhance sperm quality and fertilization rates (Fig. 5). Among these, 4 out of 19 trials are related to evaluation of the ZyMōt sperm separation technology in comparison with density gradient centrifugation. All are currently recruiting participants. A completed study by the University of California, San Francisco (NCT03085433) demonstrated that the microfluidic sperm sorting approach significantly improved embryo quality, by reducing sperm DNA fragmentation. Other ongoing trials, such as NCT04997070, are also evaluating the efficacy of microfluidic treatment sperm preparation. A study by Maltepe University (NCT04962074) examined the effectiveness of microfluidic sperm sorting in cases of reduced sperm motility, while NCT05693220 investigated the use of the ZyMot™ Multi sperm separation device for unexplained infertility and male factor infertility. One study (NCT03355937) is investigating the effectiveness of microfluidic sperm separation in couples facing unexplained infertility or repeated implantation failures. However, despite promising results, consistent evidence across diverse patient populations are needed to establish the superiority of these new approaches over traditional sperm selection methods.

**Figure 6.**
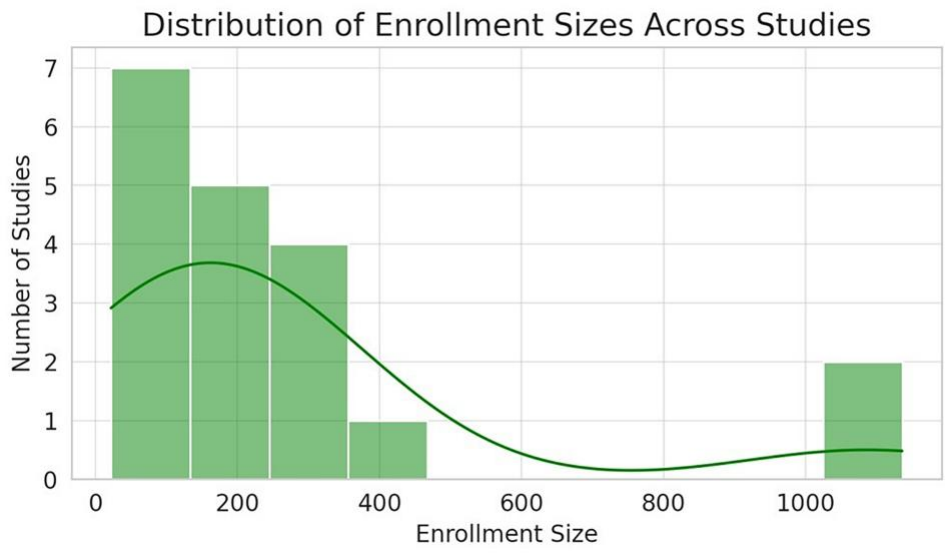
Distribution of enrolment sizes across trials: number of studies distribution vs participant enrolment size.

**Figure 7.**
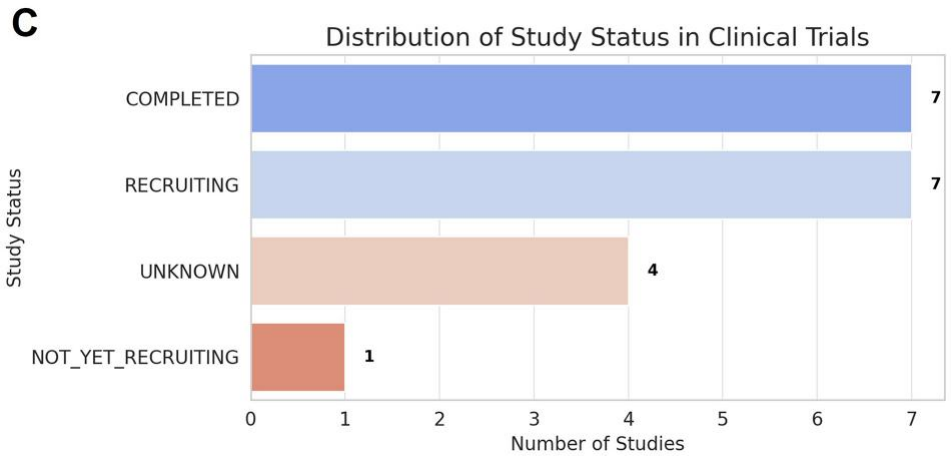
Distribution of studies’ status in clinical trials.

In alignment with this last point, as summarized in Fig. 6, the majority of these trials has a limited enrolment size and still ongoing (Fig. 7), with participant characteristics that contribute to the measurement of the performances for defined groups of patients.

Only one study conducted by the Federal University of São Paulo (NCT01003548) has compared a dynamic microfluidic embryo culture system against a traditional static culture, marking one of the earliest investigations into the advantages of this new embryo culture technology. While not directly related to ART, research on microfluidic platforms for neonatal diagnostics (NCT03291496) underscores the versatility of this technology in medical applications, including precise diagnostics and treatment monitoring. Challenges for this specific application are related to need for robust clinical evidence demonstrating their superiority over conventional methods. Regulatory bodies such as the FDA and MHRA require extensive long-term studies, including pregnancy and live birth rates, before approving these systems for routine clinical use.

### Combination with engineering innovations

The microfluidic approach can be applied to facilitate specific tasks, such as time-lapse imaging, non-invasive, dynamic sampling to measure metabolites in spent culture media, and injections. It can also be integrated and combined with complementary technologies to innovate and improve ART treatments in the next decade. One of the most promising developments in this field is the next-generation sequencing of cell-free DNA recovered non-invasively from spent blastocyst medium and used to estimate embryo chromosome copy number this avoiding the need for trophectoderm biopsy (Vera-Rodriguez *et al.*, 2018; Rubio *et al.*, 2019, 2020). The flexibility of design and microfabrication processes could also automate the sampling of the spent media at an earlier stage of embryo development while maintaining control of the culture conditions in close proximity to the embryo. Metabolic sensors embedded in microfluidic culture chambers designed with the ultimate goal of measuring oxygen, lactate, and glucose consumption (Obeidat *et al.*, 2019), could be used to quantify the stability of the culture environment and thus enhance quality control in the clinics without requiring invasive sampling. Vargas-Ordaz *et al.* (2025) recently published a study focused on the combination of light-sheet microscopy and a microfluidic device, to non-invasively image early mouse embryos. This work confirmed that high-resolution 3D, real-time imaging of live mouse embryos with minimal light exposure, maintained embryo viability, and promoted the possibility of capturing autofluorescence from NADH and NADPH and using these key metabolic cofactors to predict blastocyst formation with high accuracy (AUC 0.974) (Vargas-Ordaz *et al.*, 2025).

As they are transparent and compatible with optical microscopy and time-lapse systems (Mancinelli *et al.*, 2024a), microfluidic devices can be easily combined with AI, and machine and deep learning applied in ART (Olawade *et al.*, 2025) for assessing gamete quality (Bhide *et al.*, 2024; Shahali *et al.*, 2024) as well as for embryo annotation and selection (Fitz *et al.*, 2021). While current time-lapse systems rely on accurate positioning of early-stage embryos in spatially fixed wells in drops of media overlaid by oil, microfluidics would present a cleaner, closed, and oil-free alternative system, that limits optical aberration and artefacts thanks to their consistent thinness of the devices.

Another revolutionary approach involves 3D bioprinting, which facilitates the recreation *in vitro* of physical and chemical cues of human tissues. Microdeposition or patterning of proteins and chemo attractants, such as hyaluronic acid (West *et al.*, 2022), are achievable on plastic substrates and recent early evidence shows the possibility to adapt advanced 3D printing for deposition into micrometric channels (Mancinelli *et al.*, 2024b). Surface activation would then facilitate the combination of sperm selection microfluidics to progress towards IVF-on-a-chip.

The convergence of microfluidics with AI, time-lapse, 3D bioprinting, and metabolic monitoring can rapidly progress thanks to advancement of computing and microfabrication techniques that guarantee both safe materials and processes. The co-development of these approaches needs to involve embryologists, engineers, and manufacturers from the initial conceptualization to avoid challenges in the validation and regulatory approval processes that can stop these innovative techniques entering the clinics and improving ART treatment outcomes.

## Conclusions

The translation of microfluidic technologies from proof-of-concept to clinical application in ART remains complex, with significant technical, regulatory, and clinical challenges. Microfluidic technologies have made significant advancements in sperm processing for ART, offering improved sperm selection methods that may prove to enhance fertilization, pregnancy, and live birth rates. These systems need to successfully replicate physiological selection mechanisms, are easy to integrate into existing ART workflows, and provide higher consistency and efficiency compared to conventional methods. To date, the rapid adoption of microfluidics into ART has only happened for sperm selection and sorting and this implementation has been facilitated by minimal training requirements, disposable designs, and compatibility with other standard clinical equipment. Clinical trials continue to provide evidence of improvements in blastocyst formation, utilization, and euploidy rates following ICSI compared to traditional density gradient centrifugation methods. In contrast, progress in the uptake of microfluidic approaches for oocyte processing and fertilization has been slower due to the complexity of handling delicate oocytes, the need for precise manual skills, and the lack of clear advantages over traditional methods. Most studies remain at the pre-clinical stage, primarily using animal models, and have yet to demonstrate substantial clinical benefits that justify the increased costs or the need for additional automation. Ensuring safety and proving superior outcomes in human trials remain key challenges for widespread adoption in IVF clinics and this is difficult to achieve due to regulatory restriction and limitations on access to human samples to collect the required pre-clinical safety testing data. Despite the scientific advancements, clinical trials are needed to establish the benefits and address potential risks associated with microfluidic embryo culture systems, as well as to confirm the effectiveness of microfluidic cryopreservation methods. The proposed use of microfluidic systems for *in vitro* culture of ovarian follicles and oocytes is an exciting way to replicate the dynamic and complex environment of ovarian tissue *in vivo* which needed to support human follicle and oocyte development over extended periods. Establishment of the safety and efficacy of complete IVG systems is critical for success combined with the need to exclude materials toxicity and potential fluid-induced stresses on delicate tissues.

Despite the growing number of patents and clinical studies, securing regulatory approval remains a bottleneck, with variations in trial design requirements and complex requirements for accessing human samples and establishing robust long-term evidence of efficacy and safety.

## Data Availability

Data available on request.
